# Essential Thrombocythemia Due to Janus Kinase 2 Mutation Unmasked After Splenectomy

**DOI:** 10.7759/cureus.15357

**Published:** 2021-05-31

**Authors:** Archana Khanduri, Rahul Gupta, Jyoti Gupta, Houssem Ammar

**Affiliations:** 1 Gastrointestinal Surgery, Synergy Institute of Medical Sciences, Dehradun, IND; 2 Radiation Oncology, Swami Rama Himalayan University, Dehradun, IND; 3 Surgery, Sahloul Hospital, Sousse, TUN

**Keywords:** thrombocytosis, jak 2 mutation, splenectomy, essential thrombocythemia, splenic trauma, myeloproliferative disorders

## Abstract

Reactive thrombocytosis after splenectomy is common and often self-limiting. However, thrombocytosis can be multifactorial, especially extreme thrombocytosis (platelet count > 100 x 10^4^/cubic mm). It can lead to thrombotic or hemorrhagic complications. Hence, in patients with rising platelet count after splenectomy, detailed evaluation may be required to rule out other causes of thrombocytosis, such as infection, iron deficiency, and myeloproliferative disorders. Timely treatment of patients with thrombocytosis can prevent the development of life-threatening complications. The index case highlights the importance of regular follow-up of the patients after splenectomy to detect thrombocytosis and suspect other causes if the spleen was diseased or the platelet count fails to resolve spontaneously.

## Introduction

Thrombocytosis, defined as a platelet count above 450 x 10^9^/L, is frequently encountered in clinical practice. Generally, it is either primary or secondary. Primary thrombocytosis is characterized by increased production of platelets in the bone marrow due to myeloproliferative disorders, such as chronic myeloid leukemia, polycythemia vera, essential thrombocythemia (ET), and myelodysplastic syndrome [1]. Secondary or reactive thrombocytosis is caused by various conditions, such as anemia (due to blood loss, iron deficiency), infection, neoplasm, and splenectomy [2].

Splenectomy is one of the leading causes of thrombocytosis [3]. Thrombocytosis after splenectomy is observed in about 75%-82% cases [3]. The platelet count peaks around 1-3 weeks after splenectomy and subsequently normalizes within weeks to months [3]. The common complications of thrombocytosis include thrombosis and hemorrhage [3,4]. The differentiation between primary and reactive thrombocytosis is essential as the thrombotic and hemorrhagic complications are more frequent in primary thrombocytosis than reactive thrombocytosis [1]. However, several laboratory investigations including cytogenetic studies and bone marrow examination are required to distinguish between them. We report a case of extreme thrombocytosis (EXT) occurring after emergency splenectomy for splenic trauma. Initially, it was assumed to be reactive thrombocytosis, but on cytogenetic analysis, the patient was found to have ET due to Janus kinase 2 (JAK2) (V617F) gene mutation.

## Case presentation

A 24-year-old male presented in the emergency department after 20 hours of a road traffic accident with blunt abdominal injury. After the accident, the patient experienced abdominal pain, vomiting, and chest pain. He had no history of loss of consciousness, bleeding from the ear, nose, and throat, or seizure. He had no significant past medical and surgical history. He had a history of alcohol intake. On clinical examination, the patient was drowsy, afebrile, and had tachycardia with normal blood pressure. Abdominal examination revealed diffuse abdominal distension. Contrast-enhanced CT of the abdomen revealed splenic laceration with 6 x 6.7 x 5.5 cm splenic hematoma suggestive of American Association for the Surgery of Trauma Grade III splenic injury with moderate hemoperitoneum. The laboratory parameters on admission have been listed in Table 1.

**Table 1:** Trends in the platelet count of the patient during the course of treatment

PARAMETERS	ON ADMISSION	PREOPERATIVE (D-0)	POSTOPERATIVE (D-0)	POSTOPERATIVE (D-42)	POSTOPERATIVE (D-70)	POSTOPERATIVE (D-96)
Hemoglobin (g/dL)	9.6	7.9	7.9	13.0	13.6	13.2
Hematocrit (%)	29.6	24.8	24.2	42.0	44.9	42.9
Total leucocyte count (/cubic mm)	27,000	20,150	23,130	10,060	12,390	8,530
Platelet count (x10^9^/L)	619	468	390	1,319	1,464	702
Neutrophil count (%)	89	90	85	62	61	51
Lymphocyte count (%)	08	10	15	32	33	41

Initially, the patient was managed conservatively with fluid resuscitation as he was hemodynamically stable on admission. However, because of a fall in hemoglobin and persistent tachycardia, the patient underwent emergency splenectomy. Intraoperatively, about 1.5 L of blood and blood clots were present in the peritoneal cavity. The spleen was enlarged with multiple deep lacerations and active bleeding. The rest of the visualized viscera were unremarkable. The operative time was 75 minutes, and the estimated blood loss was around 600 mL. He was transfused 4 units of packed red blood cell in the perioperative period. Postoperative recovery was uneventful. On gross examination, the resected spleen measured 13 x 11 x 6.5 cm and weighed 520 g with multiple capsular tears involving the splenic hilum. On microscopic examination, the splenic parenchyma was unremarkable. The patient was discharged on postoperative day 6 on a normal diet. On follow-up, the patient was asymptomatic. Routine blood investigation after six weeks of surgery revealed thrombocytosis (Table 1). With the provisional diagnosis of reactive thrombocytosis, he was started on low-dose aspirin (150 mg once a day). Repeat blood count after one month showed rising platelet count to 146.4 x 10^9^/L (Table 1). Hematology consultation was taken to rule out other causes of thrombocytosis. Detailed investigation revealed JAK2 V617F/G1849T gene mutation on qualitative real-time polymerase chain reaction (Table 2). The patient was started on hydroxyurea (1,000 mg/day). Repeat complete blood count after two weeks showed a significant reduction in platelet count (Table 1). At the last follow-up, the patient was asymptomatic without any side effects of hydroxyurea.

**Table 2:** The results of genetic testing for myeloproliferative disorders BCR-ABL, fusion gene on Philadelphia chromosome; CALR, calreticulin; JAK2, Janus kinase 2; MPL, myeloproliferative leukemia; PCR, polymerase chain reaction.

PARAMETERS	RESULTS
CALR mutation (PCR, sequencing)	Not detected
JAK2 exon 12 mutation (PCR, fragment analysis)	Not detected
MPL mutation (PCR, fragment analysis)	Not detected
JAK2 V617F gene mutation (qualitative real-time PCR)	Detected
BCR-ABL gene rearrangement	Negative

## Discussion

Reactive thrombocytosis is the most common cause of thrombocytosis [2]. Various studies have reported reactive thrombocytosis to be present in 70%-80% cases and primary thrombocytosis in 20%-30% of them [1,2]. EXT, as seen in the index case, is defined as platelet count >100 x 10^9^/cubic mm [5]. In a study of 280 patients with EXT by Buss et al., reactive thrombocytosis was the main cause in more than 80% cases and myeloproliferative disorders was present in only 14% cases [6]. The main etiologies of extreme reactive thrombocytosis in their study included infection (31%), splenectomy (19%), malignancy (14%), and trauma (14%) [6]. In the present case, the combination of JAK2 mutation and splenectomy led to EXT.

While thrombocytosis is mostly asymptomatic, high platelet count may cause symptoms and signs, such as headache, vasomotor phenomenon, visual disturbances, fatigue, chest pain, abdominal pain, vertigo, aphasia, and dysarthria independent of the etiological causes [7]. In reactive thrombocytosis after splenectomy, the platelet count peaks at one to three weeks and gradually returns to normal range in few months and rarely years [3,4].

Thrombocytosis can cause life-threatening complications, such as hemorrhage and thrombosis. Post-splenectomy venous thrombosis is usually associated with platelet count more than 600 x 10^9^/L to 800 x 10^9^/L and occurs in approximately 5% of patients [8,9]. Moreover, post-splenectomy thrombocytosis can result in arterial thrombosis leading to stroke or myocardial infarction [9,10].

The first step in the management of thrombocytosis is to determine whether it is a primary process or a reactive response. In reactive thrombocytosis, the platelet count is expected to normalize after resolution of the underlying condition. In most of the cases, thrombocytosis resolves in four weeks. In a primary process such as ET, there is persistent elevation in the platelet count. Moreover, these patients carry a higher risk of thrombotic complications, especially in those with advanced age (>60 years), previous history of thrombotic episode, hypercholesterolemia, and cigarette smoking [11]. Our patient had asymptomatic thrombocytosis preoperatively, which worsened after splenectomy. Although the patient did not develop any thrombotic or hemorrhagic complications, detailed investigations for EXT unmasked the presence of JAK2 (V617F) gene mutation. JAK2 mutation is present in about 55%-60% cases of ET as seen in the present case. Other mutations observed in ET include calreticulin (15%-30%) and myeloproliferative leukemia (1%-5%) genes. About 20% patients may have “triple-negative” ET [11,12].

The major complications of ET are hemorrhage, myelofibrosis, and leukemic transformation [3]. The rate of progression to myelofibrosis is 4%-11% [13]. The long-term survival of patients with ET was shorter compared to the sex- and age-adjusted control population with the median survival of 20 years [14]. 

The management of the thrombocytosis mainly focuses on the treatment of the underlying cause and prevention of thrombotic complications. The pharmacologic agents used to reduce the risk of thrombotic complications include low-dose aspirin, ticlopidine, and enoxaparin [3,12]. The most commonly used cytoreductive agents to reduce platelet count are hydroxyurea, anagrelide, busulfan, and interferon-alpha [12]. As per the current guidelines, the patients with ET are stratified based on the risk of thrombosis into four groups: very low-risk, low-risk, intermediate-risk, and high-risk disease [12]. Aspirin alone is indicated for patients with very low-risk and low-risk disease [12]. Hydroxyurea in combination with aspirin is used in patients with intermediate- and high-risk disease. Second-line drugs, such as anagrelide, interferon-alpha, and busulfan, are used in hydroxyurea-intolerant or -refractory patients [12].

## Conclusions

Reactive thrombocytosis after splenectomy is very common but EXT is rare. EXT is most often multifactorial. Hence, patients having EXT after splenectomy should be evaluated to rule out underlying infection, iron deficiency, or myeloproliferative disorder. Also, patients with enlarged or diseased spleen undergoing splenectomy should be investigated for myeloproliferative disorder. Treatment of ET depends on the risk of thrombotic complications based on the age, smoking habit, previous thrombotic episodes, and presence of cardiovascular risk factors such as hypercholesterolemia. Aspirin and hydroxyurea are the most commonly used drugs for the treatment of ET.
